# Binding of ATP to vascular endothelial growth factor isoform VEGF-A_165 _is essential for inducing proliferation of human umbilical vein endothelial cells

**DOI:** 10.1186/1471-2091-12-28

**Published:** 2011-05-27

**Authors:** Ronald E Gast, Simone König, Karsten Rose, Katja B Ferenz, Josef Krieglstein

**Affiliations:** 1 Institut für Pharmazeutische und Medizinische Chemie, Hittorfstraße 58-62, 48149 Münster, Germany; 2 Integrierte Funktionelle Genomik, Interdisziplinäres Zentrum für Klinische Forschung, Röntgenstraße 21, 48149 Münster, Germany

## Abstract

**Background:**

ATP binding is essential for the bioactivity of several growth factors including nerve growth factor, fibroblast growth factor-2 and brain-derived neurotrophic factor. Vascular endothelial growth factor isoform 165 (VEGF-A_165_) induces the proliferation of human umbilical vein endothelial cells, however a dependence on ATP-binding is currently unknown. The aim of the present study was to determine if ATP binding is essential for the bioactivity of VEGF-A_165_.

**Results:**

We found evidence that ATP binding toVEGF-A_165 _induced a conformational change in the secondary structure of the growth factor. This binding appears to be significant at the biological level, as we found evidence that nanomolar levels of ATP (4-8 nm) are required for the VEGF-A_165_-induced proliferation of human umbilical vein endothelial cells. At these levels, purinergic signaling by ATP *via *P2 receptors can be excluded. Addition of alkaline phosphate to cell culture lowered the ATP concentration in the cell culture medium to 1.8 nM and inhibited cell proliferation.

**Conclusions:**

We propose that proliferation of endothelial cells is induced by a VEGF-A_165_-ATP complex, rather than VEGF-A_165 _alone.

## Background

Vascular endothelial growth factor isoform VEGF-A_165 _is a primarily endothelial cell-specific mitogen that plays a pivotal role in both vasculogenesis and angiogenesis [1,2]. As a key regulator of neovascularization it promotes embryonic development, wound healing and female reproductive functions [3-5]. The function of VEGF-A_165 _is associated with various medical disorders, including tumor growth and metastasis, proliferative retinopathies and inflammatory conditions such as rheumatoid arthritis and psoriasis [6-9].

There are at least eight different splice forms of the VEGF-A gene with VEGF-A_121_, VEGF-A_165 _and VEGF-A_189 _being the most abundantly expressed in humans [10-14]. All VEGF-A isoforms encode homodimeric proteins that are glycosylated and secreted. Signaling occurs through binding to the VEGF receptor 1 (Flt-1) and 2 (KDR), two structurally related receptor tyrosine kinases [15,16]. The splice forms of VEGF-A have varying affinity for heparan sulfate proteoglycans (HSPGs), depending on the different heparin-binding domains encoded by exons 6 and 7 [17-19]. The splice variant VEGF-A_165 _is thought to be most effective mitogen due to moderate heparin affinity encoded by the heparin binding domain of exon-7. This domain also facilitates the binding of VEGF-A_165 _to neuropilin 1, a co-receptor which itself enhances binding of VEGF-A_165 _to VEGFR2 [20,21].

Binding to ATP has been shown to be important for a number of growth factors, including nerve growth factor (NGF), fibroblast growth factor-2 (FGF-2) and brain-derived neurotrophic factor (BDNF) [22,23]. For BDNF, at least, this appears to be mediated by covalent binding, based on the results from mass spectrometry of BDNF-ATP complex with electrospray ionization (ESI) techniques. Other growth factor-ATP complexes were not stable under these ionization conditions, however have been detected using a more gentle ionization method, matrix assisted laser desorption/ionization (MALDI).

There is also evidence that the interaction of these factors with ATP is important for their bioactivity. For example, an interaction with ATP was proven to be a prerequisite for the neuroprotective activity of NGF and FGF2 [24,25]. Additionally, binding to ATP stabilized FGF-2 against proteolytic cleavage and thermal denaturation [26]. Although in many cases the ATP binding site and effect on protein structure is unkown, for NGF and FGF-2 at least, the nucleotide binding is thought to occur at the site of the heparin binding domain [25,27].

ATP levels are important for the nervous and vascular systems and are known to act synergistically with VEGF-A_165 _on endothelial cells [28-31]. In this study, we investigated the hypothesis that the bioactivity of VEGF-A_165 _is dependent on ATP-binding, using radiolabeling and mass spectrometry techniques. To define its biological relevance, we investigated the influence of the extracellular ATP concentration on VEGF-A_165_-induced proliferation of human umbilical vein endothelial cells (HUVECs).

## Methods

### Materials

Adenosine-5'-triphosphate (ATP) disodium salt, alkaline phosphatase (AP; from bovine intestinal mucosa), benzamidine hydrochloride, dithiothreitol (DTT), heparin sodium salt (from bovine intestinal mucosa), imadazole, lysozyme (from chicken egg white), PMSF, plasmin (from human plasma) and Triton^®^-X 100 were purchased from Sigma-Aldrich (Taufkirchen, Germany). Sodium chloride and urea were from Merck (Darmstadt, Germany), Tween^®^20 and ethylenediamine tetraacetic acid (EDTA) disodium salt from SERVA (Heidelberg, Germany), Tris-HCl from USB (Cleveland, OH, USA) and guanidine hydrochloride from GERBU (Gaiberg, Germany).

### Production and purification of recombinant human VEGF-A_165_

Heterologous expression of the plasmid pET16b-VEGFA165 in *E. coli *BL21(DE3) yielded recombinant human VEGF-A_165 _(186 aa) comprising the N-terminal His-tag sequence, GlyHis_10_, followed by a Factor Xa cleavage site. Here, the translational product is referred to as VEGF-A_165_. The expression of the plasmid was performed as described previously [27]. It resulted in the formation of inclusion bodies which represented the primary source of the target protein. They were isolated and solubilized. To that end, frozen cell pellets of *E. coli *BL21(DE3) pET16b-VEGFA165 were resuspended (Ultraturrax T 25; Jahnke & Kunkel, Staufen, Germany) in lysis buffer (0.1 M Tris-HCl pH 7.5, 5 mM EDTA, 150 mM NaCl) containing lysozyme (0.1% (w/v)), phenylmethylsulfonyl fluoride and benzamidine (1 mM each). Following sonication on ice Triton^®^-X 100 (2% (w/v)), MgCl_2 _(1 mM) and DNase I (1 μL/mL) were added for 30 min of incubation at 25°C. Subsequently, inclusion bodies were collected by centrifugation (47.800 × g, 15 min, 4°C) and washed twice with buffer (0.1% (v/v) Tween^® ^20, 150 mM NaCl) and double-destilled water (ddH_2_O) prior to solubilisation in 8 M urea, 50 mM Tris-HCl (pH 8) and 20 mM 2-mercapotethanol. VEGF-A_165 _was purified from this solution by immobilized metal ion chromatography. For that purpose, solubilized inclusion bodies were applied to an Econo-column (BioRad, Hercules, CA, USA) filled with Ni^2+^-nitrilotriacetic acid agarose (Qiagen, Hilden, Germany), washed and eluted using 250 mM imadazole according to the manufacturer's instructions. For renaturation, pooled fractions of VEGF-A_165 _were reduced with DTT (20 mM) and dialysed against renaturation buffer (500 mM guanidine hydrochloride, 100 mM Tris-HCl pH 9.0, 2 mM EDTA, cysteine/cystine redox system (5:1 ratio; 5 mM cysteine, 1 mM cystine)). Finally, refolded, dimeric VEGF-A_165 _was dialyzed against 100 mM sodium acetate buffer (pH 5) and concentrated using Amicon Ultra centrifugal filter devices (10 kDa molecular weight cut-off; Millipore, Bedford, MA, USA).

### Labeling of VEGF-A_165 _with [γ-^32^P]ATP and [α-^32^P]ATP

For labeling, 3 μg VEGF-A_165 _(unless otherwise noted) was incubated with 5 μCi each of [γ-^32^P]ATP or [α-^32^P]ATP (Hartmann Analytic, Braunschweig, Germany) and combined with 0.01 mM non-radioactive ATP (optionally containing 0.1 mM MgCl_2_). Incubation was performed in 25 mM Tris-HCl (pH 7.5, total volume 15 μL, 37°C, 15 min). For treatment of labeled VEGF-A_165 _with heparin (1, 10 or 100 μg/mL), sodium chloride (100 mM) or AP (300 ng/15 μL) incubation was continued for additional 15 min upon addition of each compound. Proteins were separated by reducing sodium dodecyl sulfate polyacrylamide gel electrophoresis (SDS-PAGE; 17.5%). Minigels were vacuum-dried. Radiolabeling was detected using a BAS-1800 II reader and BAS-MS 2325 imaging plates (Fujifilm, Tokyo, Japan) and analyzed with AIDA Image Analyzer software (version 3.21.001, Raytest GmbH, Straubenhardt, Germany).

### Plasmin digestion of VEGF-A_165 _labeled with ATP

2 μg (6 μM) VEGF-A_165 _was incubated in the presence or absence of 20 μM ATP in 25 mM Tris-HCl (pH 7.5) at 37°C for 15 min. Subsequently, 300 ng plasmin was added for proteolytic cleavage and incubation was continued for additional 120 min. For comparison with non-digested growth factor, 2 μg VEGF-A_165 _were treated in the same way except that ATP and plasmin were omitted. Protein fragments were separated by reducing SDS-PAGE (17.5%) and visualized by silver staining [32].

### Circular dichroism (CD) spectroscopy

Far-UV CD spectra (195-250 nm) were recorded at 100 nm/min using a Jasco-J600 spectropolarimeter and a 0.1 cm sample cell (25°C, 4 accumulations each). Data point resolution and bandwidth were set to 1 nm, sensitivity to 50 mdeg. Samples containing 40 μM VEGF-A_165 _were pre-incubated with or without a twofold excess of ATP (80 μM) in 25 mM Tris-HCl (pH 7.5, 37°C, 15 min). Final protein CD spectra were background-corrected with regard to absorption caused by ATP and buffer components. Data were evaluated using J-700 for Windows Standard Analysis software.

### MALDI-TOF MS

For detection of the VEGF-A_165_-ATP complex, MALDI-TOF MS was performed according to König *et al. *[23] with slight modifications. VEGF-A_165 _and its complexes were purified by reversed phase chromatography using C_18_-ZipTip pipet tips (Millipore, Bedford, MA, USA). Pipet tips were washed with elution solvent (80% methanol, 0.1% acetic acid) and equilibrated with aqueous solvent (5% methanol, 0.1% acetic acid) before use. For purification, samples containing 27.5 μM VEGF-A_165_, optionally combined with ATP (30 μM) and MgCl_2 _(60 μM), were applied, rinsed with aqueous solvent and eluted into 5 μL of elution solvent. Purified samples (0.5 μL) were spotted onto a MALDI-target coated with 0.5 μL of 1% sinapinic acid in acetone. Subsequently, 0.5 μL of 1% sinapinic acid in 40% acetonitrile was added. Spectra were obtained with MALDImicroMX (Waters Corp., Manchester, UK).

### Cell proliferation assay

HUVECs (Promocell, Heidelberg, Germany) were seeded in 96-well plates at 8 × 10^4 ^cells/well containing 100 μL of Endothelial Cell Growth Medium with supplements (EGM; Promocell, Heidelberg, Germany). Seeded HUVECs were cultured under standard conditions (humidified atmosphere, 5% (v/v) CO_2_, 37°C) for 24 h before EGM was replaced by 100 μL Endothelial Cell Basal Medium (EBM; Promocell, Heidelberg, Germany) containing 0.1% (w/v) bovine serum albumin (BSA). After 1 h of incubation, media were replaced once more by 100 μL EBM containing VEGF-A_165 _(20 ng/mL) instead of BSA. In addition, AP (Sigma-Aldrich, Taufkirchen, Germany) was applied to selected samples at 40, 80 or 160 ng/mL along with thegrowth factorand incubation was continued for 48 h. Subsequently, cell culture media were taken from separate samples treated in the same manner for measurement of extracellular ATP.

Finally, proliferation of HUVECs was determined using the CellTiter 96^® ^Aqueous One Solution Cell Proliferation Assay kit (Promega, Mannheim, Germany) according to the manufacturer's instructions. To that end, 20 μL of CellTiter 96^® ^Aqueous One Solution was added to each well to be incubated for additional 3 h. The number of viable cells was directly proportional to the absorbance of a colored formazan product determined colorimetrically (Lambda Scan, MWG Biotech, Ebersberg, Germany) at 490 nm. Values are presented as means ± standard deviation (SD; n = 9).

### Luminometric measurement of extracellular ATP

Extracellular ATP was determined in cell-free samples of cell culture media (80 μL each) employing the ATP Kit SL (sensitivity: 10^-6^-10^-12 ^mol/L; BioThema, Handen, Sweden) according to the manufacturer's instructions. The bioluminescent reaction is based on the luciferase-catalyzed oxidation of luciferin in the presence of oxygen and ATP. Besides AMP, pyrophosphate and carbon dioxide, oxiluciferin is produced emitting light at 560 nm to be measured luminometrically. Light emission is proportional to the amount of ATP and was measured in a two-step procedure using an FB 12 Single tube Luminometer (Berthold Detection Systems, Pforzheim, Germany). First, the light intensity of the samples (I_smp_) was quantified as relative light units/second (RLU/s). Then, an internal ATP standard (final concentration 10 μM) was added to each sample and light intensity was quantified again to give I_smp+std_. This procedure allowed for the conversion of light intensity (RLU/s) to concentration of ATP (mol/L) by the following equation:

The assay was performed in triplicate.

### Statistical analysis

Data are expressed as means ± SD based on one-way analysis of variance (ANOVA) followed by Scheffé's test. A probability value (P) of less than 0.01 was considered statistically significant. Figure legends specify statistically significant differences between experimental groups at probability values of p < 0.01 and p < 0.001. Analysis was performed using WinSTAT.

## Results

### Binding of ATP to VEGF-A_165_

**Figure 1:**
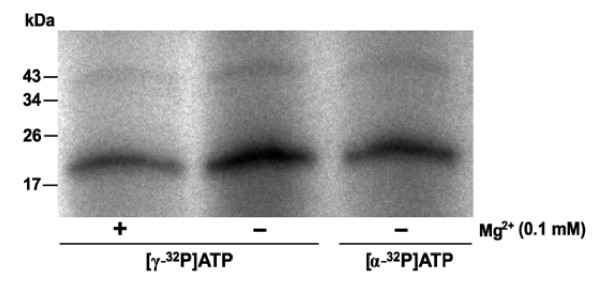
**Labeling of VEGF-A**_**165 **_**with [γ-**^**32**^**P]ATP and [α-**^**32**^**P]ATP**. VEGF-A_165 _(2 μg) was incubated with radioactive ATP (5 μCi) in Tris-HCl (pH 7.5) at 37°C. MgCl_2 _(0.1 mM) was added prior to ATP (+). After 15 min of incubation SDS-PAGE and autoradiography were performed.

**Figure 2:**
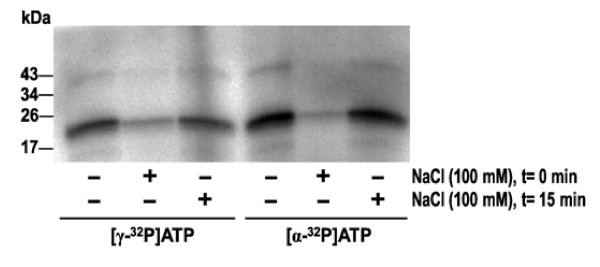
**Effect of increased ionic strength on labeling of VEGF-A**_**165 **_**with [γ-**^**32**^**P]ATP and [α-**^**32**^**P]ATP**. VEGF-A_165 _(3 μg) was incubated with radioactive ATP (5 μCi) in Tris-HCl (pH 7.5) at 37°C for 30 min. NaCl (100 mM) was added at times (t) indicated.

In order to evaluate the binding of ATP to VEGF-A_165_, VEGF-A_165 _was radiolabeled by [γ-^32^P]ATP and [α-^32^P]ATP. The use of [γ-^32^P]ATP as well as [α-^32^P]ATP allowed to distinguish between binding of ATP to VEGF-A_165 _and autophosphorylation. The influence of divalent cations (Mg^2+^) was also tested. Signal is detected for both [γ-^32^P]ATP- and [α-^32^P]ATP-labeled growth factor independently of the presence of Mg^2+ ^(Figure 1). ATP appeared to be bound to growth factor by non-covalent interaction *via *the phosphate residues of the nucleotide [23,25,27]. In contrast to a covalent modification, an ionic interaction can be influenced by an increase in ionic strength. Labeling of VEGF-A_165 _with [γ-^32^P]ATP and [α-^32^P]ATP, respectively, was suppressed by 100 mM NaCl added to the reaction mixture prior to the nucleotides (Figure 2). Once the complex had formed, however, it proved to be fairly resistant to the salt concentration.

**Figure 3:**
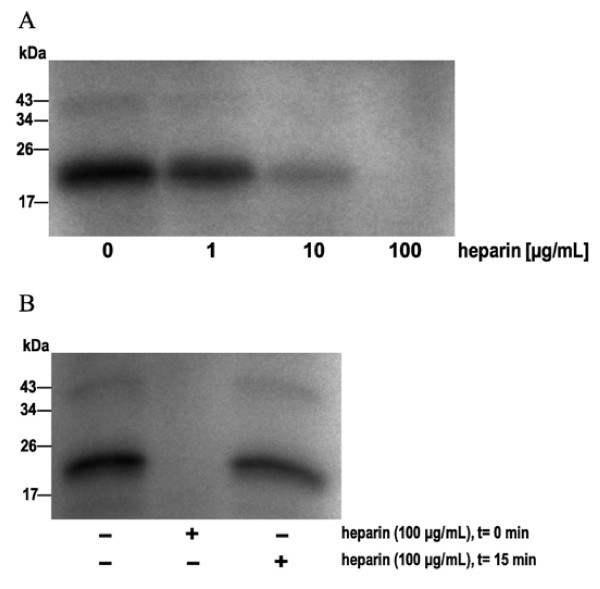
**Competition of heparin and [γ-**^**32**^**P]ATP for binding to VEGF-A**_**165**_. VEGF-A_165 _(3 μg) was labeled with [γ-^32^P]ATP (5 μCi) in Tris-HCl (pH 7.5) at 37°C for 30 min. **(A) **Heparin was added prior to ATP to give final concentrations of 1 to 100 μg/mL. **(B) **Heparin was added at a fixed concentration of 100 μg/mL and times (t) indicated.

VEGF-A_165 _contains a heparin-binding domain which is critical for its mitogenic activity and storage in the extracellular matrix *via *HSPGs [33]. Interestingly, heparin affected binding of ATP to FGF-2 due to overlapping binding sites [27]. Competition experiments revealed that heparin also interfered with the binding of ATP to VEGF-A_165_. 10 μg/mL heparin added to the reaction mixture prior to [γ-^32^P]ATP reduced radiolabeling of VEGF-A_165 _markedly (Figure 3A). 100 μg/mL heparin inhibited [γ-^32^P]ATP binding to the mitogen completely. However, when [γ-^32^P]ATP was added to the reaction mixture prior to heparin (100 μg/mL), only a slight decrease in radiolabeling of VEGF-A_165 _occurred (Figure 3B, lane 3).

### MALDI-TOF MS of the VEGF-A_165_-ATP complex

MALDI-TOF MS was performed employing soft conditions as described previously [23]. This approach was suitable for the detection of labile nucleotide-protein complexes. Sample preparations using low-acidic reversed phase chromatography and acid-free matrix assisted in retaining the non-covalent interaction. The measurements were performed using the high-mass detector in order to observe the VEGF-A_165 _dimer as the bioactive species present *in vivo *[1].

**Figure 4:**
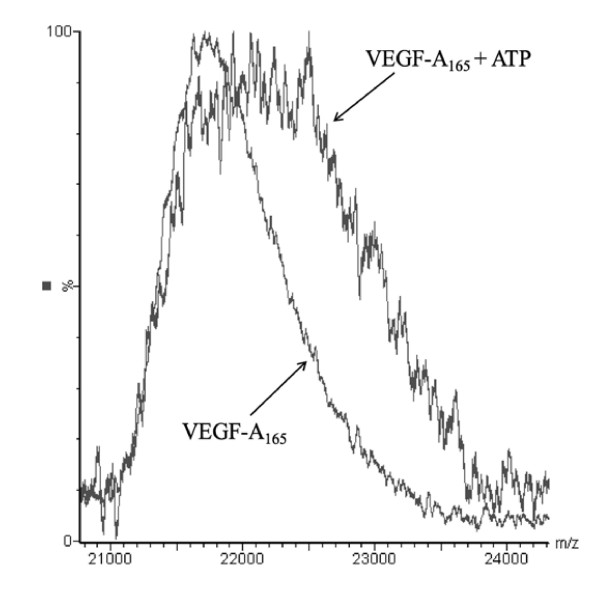
**MALDI-TOF spectrum overlay of VEGF-A**_**165 **_**and the VEGF-A**_**165**_**-ATP complex**. VEGF-A_165 _(27.5 μM), ATP (30 μM ATP). ATP (507.2 g/mol) causes peak broadening due to complex formation with VEGF-A_165_. The further addition of 60 μM Mg^2+ ^has no measurable impact on the spectrum (data not shown).

The incubation of VEGF-A_165 _with ATP (507.2 g/mol) caused considerable peak broadening as compared to pure VEGF-A_165 _(21673 Da) (Figure 4). The shift in mass corresponded to the addition of one molecule of ATP per molecule of growth factor and did not differ when Mg^2+ ^was added to the incubation mixture. The dimer was also affected by ATP, but the number of bound ATP molecules could not be clearly defined due to low signal intensity.

### ATP induces a conformational change of VEGF-A_165_

**Figure 5:**
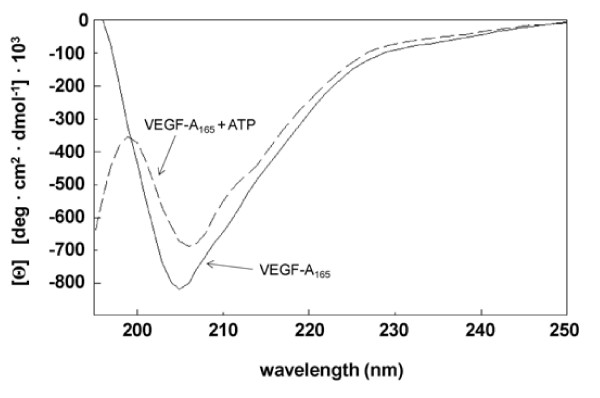
**ATP induces a conformational change in the secondary structure of VEGF-A**_**165**_. Far-UV CD spectra were recorded of VEGF-A_165 _(40 μM) pre-incubated without or with ATP (80 μM). Pre-incubation was performed in Tris-HCl (pH 7.5) at 37°C for 15 min.

Far-UV CD spectroscopy was carried out in order to investigate a putative effect of ATP binding on the secondary structure of VEGF-A_165_. Thus, the CD of VEGF-A_165 _was measured without or with a twofold molar excess of ATP. The graph obtained for (His)_10_-tagged rhVEGF-A_165 _(Figure 5) was similar to that reported in the literature for untagged rhVEGF-A_165 _reflecting a typical graph of a protein rich in β-structure [34,35]. The latter accounts for about 40% of the secondary structure of rhVEGF-A_165_. A first maximum close to the base level below 200 nm fell to a minimal molar ellipticity at 207 nm and rose again to a second maximum at 250 nm. In contrast, the CD spectrum of VEGF-A_165 _with ATP exhibited a significant decrease in molar ellipticity towards the base level above 200 nm, especially at 207 nm (Figure 5, dashed line). This result suggested reduced β-sheet content in the secondary structure of the growth factor compensated for by an increase in random coil structure.

### Binding of ATP does not protect VEGF-A_165 _from plasmin cleavage

**Figure 6:**
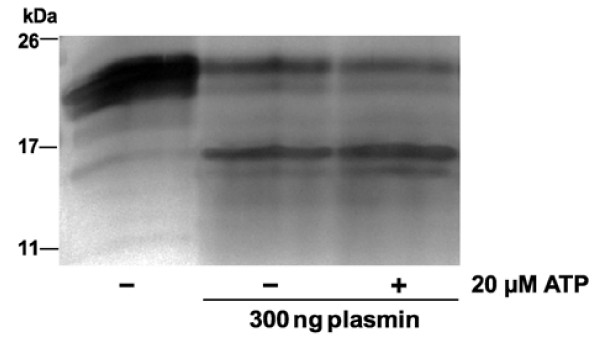
**Proteolytic cleavage of ATP-labeled and unlabeled VEGF-A**_**165 **_**by plasmin**. VEGF-A_165 _(6 μM) was preincubated in Tris-HCl (pH 7.5) without or with ATP (20 μM) at 37°C. After 15 min of pre-incubation, 300 ng of plasmin was added to one sample (lane 3) and incubation of all samples was continued for an additional 120 min. Reaction products were visualized by SDS-PAGE and silver staining.

The serine protease plasmin cleaves VEGF-A_165 _solely at the Arg^110^-Ala^111 ^bond yielding VEGF-A_110 _and an N-terminal fragment consisting of 55 residues including the heparin-binding domains [33]. Compared to VEGF-A_165_, VEGF-A_110 _exhibits a markedly reduced mitogenic activity on HUVECs. In the case of FGF-2, ATP binding protected effectively against proteolytic digestion by proteases including plasmin [26]. This was not true for VEGF-A_165 _(Figure 6). Plasmin digestion of VEGF-A_165 _and VEGF-A_165 _pre-incubated with ATP generated a most abundant cleavage product of 15 kDa in all samples which corresponded to the molecular mass of (His)_10_-tagged VEGF-A_110_.

### Mitogenic activity of VEGF-A_165 _on HUVECs requires ATP

**Figure 7:**
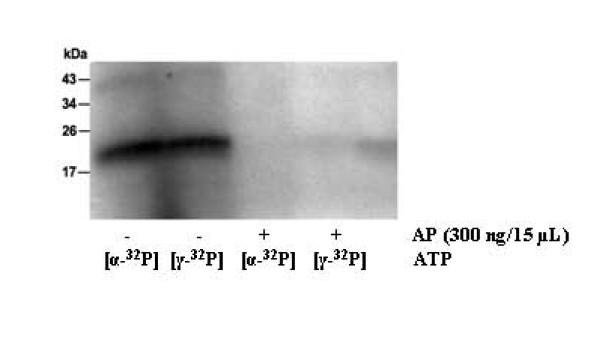
**AP hydrolyzes [γ-**^**32**^**P]ATP and [α-**^**32**^**P]ATP bound to VEGF-A**_**165**_. VEGF-A_165 _(15 μM) was labeled with radioactive ATP (5 μCi) in Tris-HCl (pH 7.5) at 37°C for 15 min. After labeling (t = 15 min), 300 ng of AP was added (lane 3 and 4). Incubation of all samples was continued for an additional period of 15 min followed by SDS-PAGE and autoradiography.

NGF and FGF-2 protected cultured neurons against damage by staurosporine only if ATP at concentrations above 1 nM were present in the culture medium [24,25]. In order to investigate this effect for VEGF-A_165_, we compared the proliferative effect of VEGF-A_165 _in untreated cultures of HUVECs with that at reduced ATP level. ATP levels in the cell culture medium were lowered by AP and measured luminometrically. AP dephosphorylated both free ATP and [γ-^32^P]ATP or [α-^32^P]ATP bound to VEGF-A_165 _(Figure 7).

**Figure 8:**
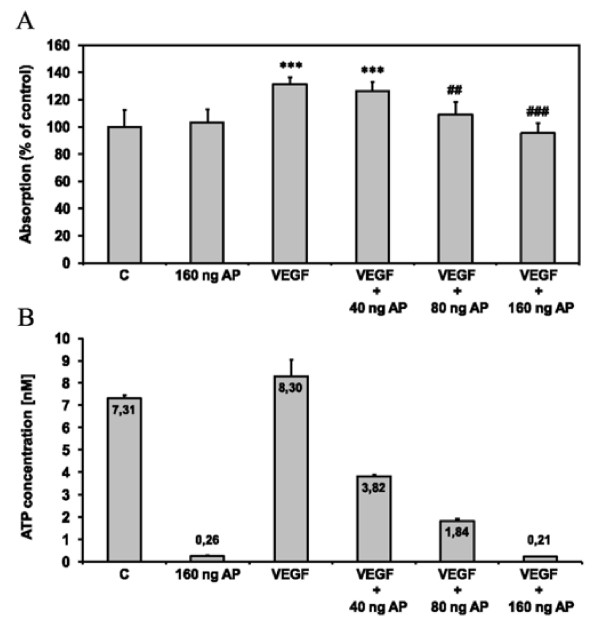
**VEGF-A**_**165 **_**fails to induce proliferation of HUVECs at low extracellular ATP concentrations**. **(A) **HUVECs were cultured in serum-free EBM (C) or serum-free EBM containing VEGF-A_165 _(20 ng/mL), AP (160 ng/mL) or VEGF-A_165 _(20 ng/mL) combined with AP at concentrations increasing from 40 ng/mL to 160 ng/mL. After 48 h of incubation CellTiter 96^® ^Aqueous One Solution Cell Proliferation Assay was performed. The absorption of a colored formazan product measured colorimetrically is proportional to the number of viable cells. Values are presented as means ± SD (n = 9). ***p < 0.001 vs. C, ^###^p < 0.001 and ^##^p < 0.01 vs. VEGF (ANOVA, Scheffé's test). **(B) **ATP concentrations corresponding to (a) were analyzed luminometrically (n = 3) in the HUVEC culture medium after 48 h of incubation.

VEGF-A_165 _(20 ng/mL) increased the number of viable HUVECs in the positive control significantly (Figure 8A, VEGF). 40 ng/mL AP reduced eATP to 3.82 nM (positive control: 8.30 nM) which did not impair the mitogenic activity of VEGF-A_165_. However, higher concentrations of AP (80 ng/mL, 160 ng/mL) inhibited HUVEC proliferation by VEGF-A_165 _due to lowering of ATP levels to 1.84 nM and 0.21 nM, respectively (Figure 8B), in the cell culture medium. AP (160 ng/mL) added without exogeneous VEGF-A_165 _did not influence HUVEC viability despite reducing eATP effectively to 0.26 nM. In line with the data obtained for NGF and FGF-2, these results proved that VEGF-A_165 _required eATP in the low nanomolar range to act mitogenically on HUVECs.

## Discussion

In previous work it has been demonstrated that growth factors such as NGF, BDNF and FGF-2 bind ATP and form non-covalent nucleotide-protein complexes [22,23] which are essential for neuroprotective activity *in vitro *[24,25]. In the case of FGF-2, binding of ATP also imparts enhanced proteolytic and thermal resistance [26]. In the present study, we detected binding of ATP to VEGF-A_165_, the predominant growth factor involved in neovascularization. Both radiolabeling (Figure 1) and mass spectrometry (Figure 4) analyses suggested that ATP bound to VEGF-A_165 _is independent of Mg^2+^-ions. The VEGF-A_165_-ATP complex appears to be extremely stable, remaining intact after denaturing SDS-PAGE, solid phase extraction and mass spectrometry techniques. In addition, an increase in ionic strength caused only a minor dissociation of the complex (Figure 2).

The most physiologically important form of ATP is thought to be the ATP/Mg^2+^-complex, which is the predominant form of the nucleotide in tissue. Although our mass spectrometry analyses provide strong evidence that ATP bound to VEGF-A_165 _independently of Mg^2+^-ions, labeling of VEGF-A_165 _with [γ-^32^P]ATP could also be observed with 0.1 mM MgCl_2 _in the reaction buffer (data not shown). This is also true for labeling of the growth factor NGF [25]. Such ATP/Mg^2+^/growth factor complexes were identified by MALDI-TOF analysis of the growth factors FGF2 and NGF recently [23]. Radiolabeling of NGF with ATP is also possible in buffers containing Ca^2+^, Mg^2+^, Mn^2+ ^or Ni^2+^, respectively [25]. Taken together, this indicates that VEGF-A_165 _also forms a complex with ATP at physiological Mg^2+^-concentrations.

Additionally, the recently discovered stabilization of FGF2 by ATP is also present when using Mg^2+^-ions [36]. This observed stabilizing effect of ATP on the growth factor is present at Mg^2+ ^concentrations of 0.1 mM. This indicates that under these conditions ATP/Mg^2+ ^binds to FGF2 and that this physiological ATP/cation complex protects FGF2 against degradation, too. The effect of ectonucleases on the VEGF-A_165_-ATP complex also remains unknown. Our results suggest that the ATP bound to VEGF-A_165 _was not only completely susceptible to cleavage by alkaline phosphatase (Figure 7), but also moderately susceptible to apyrase (data not shown).

Our results are consistent with the theory that growth factors bind ATP despite the absence of classic ATP binding site. Nevertheless, NGF, FGF-2 and VEGF-A_165 _contain heparin binding domains, characterized by clusters of basic residues [37-39], which may interact with the negatively charged phosphate residues of ATP. The removal of these basic residues by site-directed mutagenesis of NGF and FGF-2 has been shown to drastically reduced both ATP binding and neuroprotective activity [25,27,40]. Heparin has been shown to suppress the binding of ATP to VEGF-A_165 _(Figure 3), however does not cause the existing VEGF-A_165_-ATP complex to dissociate. Nevertheless, the competition between ATP and heparin for binding to VEGF-A_165 _is likely to effect the interaction with the VEGF receptor or storage in the extracellular matrix.

Our results strongly suggest that ATP binding induces a conformational change in the secondary structure of VEGF-A_165_. We propose that this conformational change is responsible for the increased bioactivity of the VEGF-_165_-ATP complex, resulting in improved ligand-receptor interaction (Figure 5). The location at which ATP binds VEGF-A_165_, as well as the exact nature of the conformational change remains unknown.

Brandner *et al. *[34] demonstrated that heparan sulfate induced a conformational change in glycosylated VEGF-A_165 _but not in the non-glycosylated form. In addition, they showed that heparin stabilized both glycosylated and non-glycosylated VEGF-A_165 _against chaotropic or thermal denaturation without inducing any conformational change. This implies that the competition of ATP and heparin (HSPGs) for binding to the mitogen is of biological relevance. Further studies have to be undertaken in order to define exactly where ATP is bound providing a basis for elucidating putative complex-receptor interactions.

We found no evidence that ATP binding protects VEGF-A_165 _against plasmin cleavage (Figure 6), as previously suggested for FGF-2 [26]. We therefore propose that the biological activity of the VEGF-A_165_-ATP complex is due to improved receptor binding, and not due to increased stability of the growth factor. Supporting this theory, VEGF-A_165 _failed to induce HUVEC proliferation when the ATP concentration in the cell culture media was too low (Figure 8). This result corresponded to the minimal concentration of eATP required for the neuroprotective activity of NGF and FGF-2, which was determined to be approximately 1 nM [24,25].

In our cell culture experiments, alkaline phosphatase was required to lower the concentration of ATP to levels that affected cell proliferation. It is feasible that high concentrations of alkaline phosphatase had independent effects, and we cannot discount the possibility of minor contamination with proteases. Alkaline phosphatase added without exogeneous VEGF-A_165 _did not influence HUVEC viability (despite reducing eATP levels to 0.26 nM; Figure 8). Therefore, we believe side effects of alkaline phosphates at these concentrations are unlikely. This is in line with investigations with other growth factors like FGF2 [24] and NGF [25] that demonstrated similar results when using alkaline phosphatase to lower eATP-concentrations.

A completely different observation was made when using another growth factor, granulocyte colony stimulating factor (GCSF). This factor does not bind ATP and the neuroprotective activity of GCSF is not influenced by degradation of extracellular ATP by alkaline phosphatase (data not shown). This is in contrast to the situation with the ATP-binding growth factors FGF2 and NGF, where an extracellular ATP-concentration above about 1 nM is essential for the neuroprotective activity of these growth factors [24,25]. However, this is plausible in the context of our hypothesis that ATP-growth factor interaction is essential for the activity of ATP-binding growth factors but not important for non-ATP-binding growth factors like GCSF. It is therefore reasonable to assume that the observed inhibition of HUVEC proliferation by VEGF-A_165 _was due to the low ATP levels caused by alkaline phosphatase, which prevented the formation of the presumed biologically active VEGF-A_165_-ATP complex.

It is known that eATP can act synergistically with angiogenic growth factors including VEGF-A_165 _*via *P2Y receptor signaling [30]. Even in the absence of VEGF-A_165_, the nucleotide itself is capable of P2Y_1/2_-VEGFR2 transactivation, inducing endothelial cell proliferation [31,41]. Given that activation of P2X and P2Y receptors requires eATP in the micromolar range [42], isolated effects of eATP mediated by purinergic receptor signaling are unlikely to contribute to the experimental results obtained from our model (Figure 8). We have clearly shown that ATP is tightly bound to VEGF-A_165 _and a critical concentration of ATP above 1.8 nM is required for bioactivity. Based on these results, the VEGF-A_165_-ATP complex and not VEGF-A_165 _by itself appears to be the active ligand causing the proliferative effects under cell culture conditions. Both VEGF-A_165_-ATP complex formation and the putative interaction with its receptor remain to be elucidated *in vivo*.

## Conclusions

For the first time we provided ample evidence that ATP binds to VEGF-A_165_. Binding of ATP most likely involves basic residues within the heparin binding domain and constitutes a prerequisite for the proliferative activity of VEGF-A_165_.

## List of abbreviations

The abbreviations used are: AP: alkaline phosphatase; eATP: extracellular ATP; EBM: endothelial cell basal medium; EGM: endothelial cell growth medium; ESI: electrospray ionization; GF: growth factor; HSPG: heparan sulfate proteoglycan; HUVEC: human umbilical vein endothelial cell; rh: recombinant human; RLU/s: relative light units/second; SDS-PAGE: sodium dodecyl sulfate polyacrylamide gel electrophoresis.

## Authors' contributions

REG conceived of, designed and carried out all experiments including statistical analysis and drafted the manuscript. SK performed all MS measurements and helped to draft the manuscript. KR participated in the CD spectroscopy analysis and helped to draft the manuscript. KBF participated in the luminometric measurement of ATP. JK initiated the study, participated in conception, design and coordination of the study and helped to draft the manuscript. All authors read and approved the final manuscript.
